# The effects of amino substituents on the enhanced ammonia sensing performance of PcCo/rGO hybrids†

**DOI:** 10.1039/c8ra07509c

**Published:** 2018-12-11

**Authors:** Bin Wang, Xiaolin Wang, Xiaocheng Li, Zhijiang Guo, Xin Zhou, Yiqun Wu

**Affiliations:** Key Laboratory of Functional Inorganic Material Chemistry, Ministry of Education, School of Chemistry and Materials Science, Heilongjiang University Harbin 150080 P. R. China wangbin@hlju.edu.cn; School of Material and Chemical Engineering, Heilongjiang Institute of Technology Harbin 150050 P. R. China; MIIT Key Laboratory of Critical Materials Technology for New Energy Conversion and Storage, School of Chemistry and Chemical Engineering, Harbin Institute of Technology Harbin P. R. China; Shanghai Institute of Optics and Fine Mechanics, Chinese Academy of Sciences Shanghai 201800 P. R. China

## Abstract

Three reversible ammonia (NH_3_) gas sensors were fabricated using tetra-α-(*p*-aminobenzyloxy)phthalocyanine cobalt (ABOPcCo), tetra-α-aminophthalocyanine cobalt (APcCo) and substituent-free phthalocyanine cobalt (FPcCo) functionalized reduced graphene oxide (rGO), with cost-efficient, highly sensitive and stable sensing performance. These hybrid materials were prepared *via* a facile physical solution mixing self-assembly reaction with rGO and PcCo solutions. The obtained PcCo/rGO hybrid sensors exhibit excellent sensing performance; especially the ABOPcCo/rGO sensor, whose response is about 23.3% (50 ppm), with a limit of detection as low as 78 ppb, and response and recovery times about as fast as 225 s and 250 s. The performance of the PcCo/rGO hybrid sensors can be optimized by adjusting the concentrations of the PcCo/rGO aqueous dispersions. More importantly, the NH_3_-sensing performance of the PcCo/rGO sensors was tuned by adjusting the substituent structure of PcCo. The enhanced NH_3_-sensing performance may be attributed to synergistic effects between PcCo and rGO, *e.g.*, stronger adsorption interactions between PcCo with an aminophenoxy substituent and NH_3_, the high electrical conductivity of rGO, and fast charge transfer between PcCo and rGO. These are further confirmed *via* first-principle density functional theory (DFT) calculations and electrochemical impedance spectra (EIS) measurements.

## Abstract 2

Three new room temperature reversible ammonia gas sensors were fabricated using PcCo/rGO hybrids with cost-efficient, highly sensitive and stable sensing performance.

## Graphical abstract

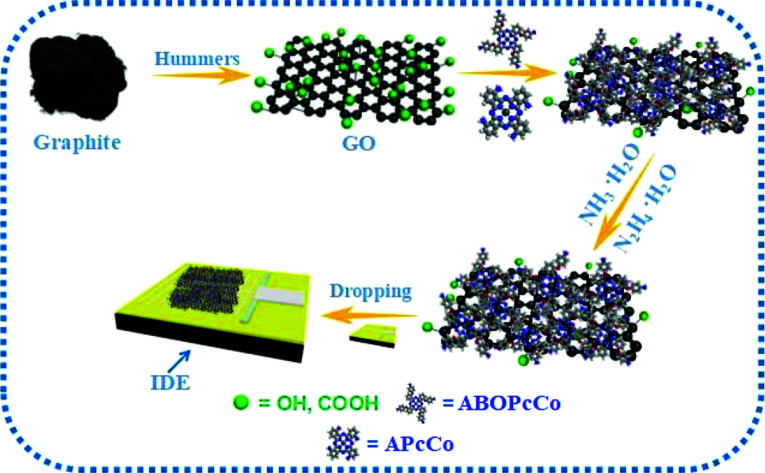

## 1. Introduction

During the last few decades, gas sensors have boosted advances in agriculture, the food industry, industrial chemicals, and environmental and security areas.^1–4^ Obtaining highly selective, sensitive, cost-efficient and stable sensing materials is one of the key points for the development of new gas sensors for environmental and human health applications. Nanocomposite materials have recently attracted extensive interest for gas sensing applications due to synergetic and complementary effects.^5–8^ Among them, graphene and its nanocomposites, with two-dimensional sp^2^-hybridized carbon atoms, are currently, without any doubt, the most attractive gas sensing materials due to their large specific surface areas and electron transport through them being highly sensitive to the absorption of gas molecules.^9–15^

In 2007, Novoselov's group reported the first graphene sensor for detecting gases.^16^ They showed that conductance changes in graphene are dependent on the nature of the gas molecules, which indicates that charge transfer between the gas molecules and graphene is responsible for the conductivity changes. This study has opened the door for a new type of gas sensor based on 2D graphene. While there are a few drawbacks relating to the limits of sensitivity and lack of selectivity with respect to graphene based sensors, defective and functionalized graphene may result in gas sensors with improved and efficient gas sensing performance, such as increased sensitivity, selectivity, response and recovery, through promoting charge transfer between graphene and the gas molecules.^17–21^ Owing to there being certain amounts of oxygen groups in reduced graphene oxide (rGO) that provide reactive sites for gas adsorption and further functionalization, rGO based gas sensors show improved sensing characteristics. Moreover, the conductance of such sensors can be restored through a chemical reduction process.^10,22,23^ The functionalization of rGO with functional materials (such as polymers, metals and metal oxide nanoparticles) has also been reported to improve its sensing efficiency.^24–30^

Metal phthalocyanines (MPcs) show improved sensing characteristics due to their 18π-conjugated skeletons, fine-tuned structures and high solubilities in solvents.^31–34^ However, the low conductivities of MPc based gas sensors have limited their practical applications.^35^ Importantly, MPcs are an ideal candidate for the functionalization of rGO, considering their possible further modification.^36–39^ The use of nanocomposites of MPcs and rGO may result in gas sensors with improved and efficient gas sensing characteristics, which may help to solve particular shortcomings through synergetic and complementary effects. Our group has reported gas sensors based on carbon nanotube (CNT)/MPc, graphene oxide (GO)/MPc and rGO/MPc hybrids.^40–42^ These sensors showed higher sensitivity toward NH_3_ than their carbon material counterparts. It was observed that the presence of functional MPcs played an important role in the sensing process. Nowadays there have been many studies reporting the effects of substituents on pristine MPcs for gas sensing,^43,44^ but few systematic studies to date have reported the effects of substituent groups on the NH_3_ sensing characteristics of rGO/MPc hybrids.^45^

**Scheme 1:**
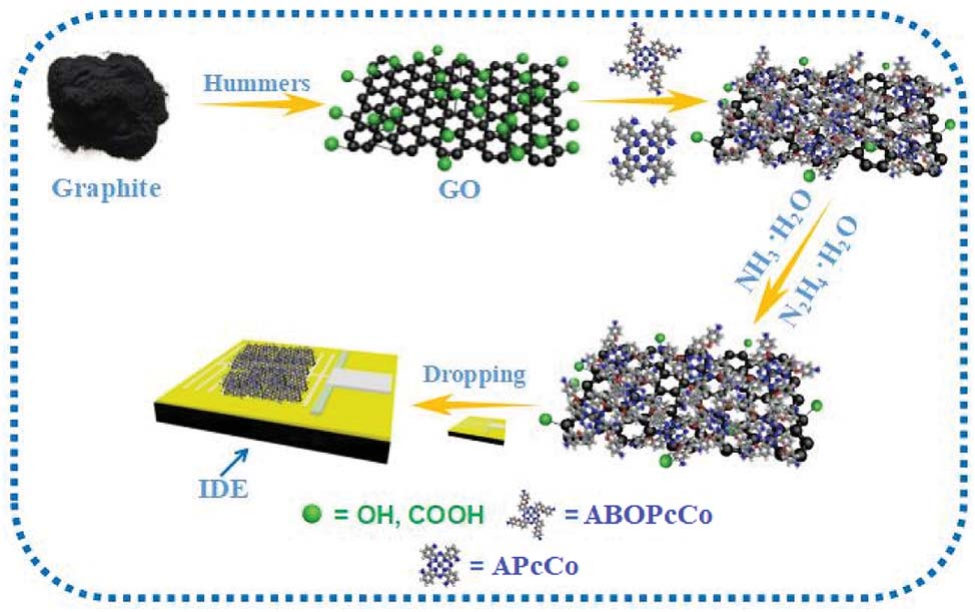
Schematic diagram showing the preparation and NH_3_-sensing of sensors composed of PcCo/rGO hybrids.

Considering the above-mentioned reasons, herein, we design three types of PcCo compound, tetra-α-(*p*-aminobenzyloxy)phthalocyanine cobalt (ABOPcCo) and tetra-α-aminophthalocyanine cobalt, (APcCo) containing different amino substituents, and substituent-free phthalocyanine cobalt (FPcCo), and combine them with rGO functionalization to develop new high performance chemiresisitor-type NH_3_ sensors based on the ABOPcCo/rGO, APcCo/rGO and PcCo/rGO hybrids (as shown in Scheme 1). The PcCo/rGO sensors show better responses, and excellent selectivity and recovery characteristics in NH_3_ sensing. The gas sensing characteristics of the rGO based gas sensors are improved considerably due to the functionalization of rGO with PcCo.

## 2. Experimental and calculation details

### 2.1 Reagents

PcCo compounds were synthesized using 1,8-diazabicyclo[5,4,0]undec-7-ene (DBU) as a catalytic agent (the detailed process is shown in the ESI†). Graphene oxide (GO) was used, as in our previous reports.^41^ Ultrapure water was obtained using a Millipore Milli-Q system (Millipore Corp. Bedford, MA, USA). Graphite was purchased from Nanjing XFNANO Materials Tech Co., Ltd. 3-Nitrophthalonitrile (99% purity) and DBU (98% purity) were purchased from Sigma-Aldrich Co. LLC. All other reagents in this work were of analytical grade and used without further purification.

### 2.2 Preparation of PcCo/rGO hybrids

Two PcCo/rGO hybrids were prepared *via* the same general method: 20 ml of 1.0 mg ml^−1^ ABOPcCo DMF green solution was added dropwise to 10 ml of 1.0 mg ml^−1^ GO DMF suspension. The mixtures were magnetically and ultrasonically stirred for 12 h under light-avoiding conditions. Then 3 ml of ammonia and 0.03 ml of hydrazine hydrate were added into the above reaction mixture, which was heated at 100 °C for 24 h. The crude product was cooled to room temperature and washed several times successively, first with boiling water, and then with ethanol and tetrahydrofuran to remove impurities, until the filtrate was clear. The resulting black product was dried *in vacuo* for 12 h at 60 °C. Other PcCo/rGO hybrids were prepared using the same method as given above.

### 2.3 Sensor assembly and sensing measurements

The gold interdigitated electrodes (IDEs) and gas sensor testing device used were as shown in our previous studies.^40,41^ PcCo/rGO hybrids were dispersed (0.5, 1.0, 1.5 and 2.0 mg ml^−1^) in DMF under ultrasonic waves for 2 h. Then, 10 μl of PcCo/rGO suspension was dropped onto the IDE gaps using a microsyringe. Sensor films were deposited after the solution was evaporated and dried in a vacuum oven for 2 h at 80 °C. The sensor resistance was adjusted by varying the concentration of the PcCo/rGO hybrid in the DMF solution. For comparison, purified rGO and PcCo sensors were also obtained *via* the above methods.

The gas sensing performances of the sensors were measured with a CUST G2 gas sensing test system (Advanced Sensor Technology Laboratory of Jilin University, China). All measurements were performed at 28 °C ± 1 °C with a relative humidity of 50% ± 2%. First, an air flow was introduced into the sensing test chamber to record a baseline. Then, target gases at certified concentrations were injected to register sensor signals. Finally, the sensor was recovered under an air flow. The humidity in the test chamber was controlled *via* changing the mixing ratio of fully humid air and dry air, and the humidity level was checked using a humidity sensor. In this experiment, we used a high purity concentration of NH_3_ gas (99.99%) mixed with air as a carrier, using the static volumetric method to obtain a low concentration. The response is defined by the relative resistance change, as follows:1
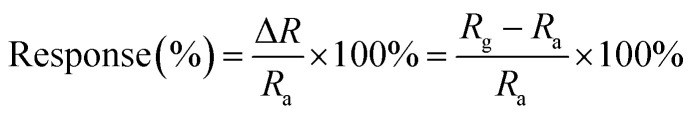
where *R*_a_ and *R*_g_ are the sensor resistance values under the initial air flow, which was used as background, and under the target gas. The response and recovery times are defined as the times needed for 90% total resistance change upon exposure to the target gas and air, respectively. High purity NH_3_ gas was used as the NH_3_ source (Guangming Research and Design Institute of Chemical Industry, PR China).

### 2.4 Characterization

UV-vis absorption spectra were recorded with a UV-2700 spectrometer (SHIMADZU, Japan). Raman spectra, acquired using Raman spectroscopy, were obtained with a Jobin Yvon HR800 Raman spectrometer, using a 458 nm laser source. Scanning electron microscopy (SEM) images were recorded with a Hitachi S-4800 field emission scanning electron microscope operating at 15 kV. Samples were drop-deposited onto the interdigitated electrodes and examined directly. Transmission electron microscopy was performed with a JEM 2100 instrument at 200 kV, utilizing a JEOL FasTEM system. Samples were dropped onto Cu grids with lacey carbon film and allowed to dry thoroughly before imaging. FT-IR spectra were recorded using a Spectrum Two spectrometer (PerkinElmer, USA). Electrochemical impedance spectra (EIS) were measured using a CHI600E electrochemical work station at room temperature (frequency range: 0.01 Hz to 100 kHz; at the open circuit potential with 5 mV amplitude).

### 2.5 Calculation details

DFT calculations were performed for the adsorption of NH_3_ on PcCo compounds using the long-range corrected functional of CAM-B3LYP with a set of hybrid basis sets (LanL2DZ for metals and 6-31g(d) for H, C, and N).^46^ Charge analyses were performed using the NBO (natural bond orbital) method.^47^ The Gaussian 09 quantum chemical package was employed for all calculations in the present work.^48^

## 3. Results and discussion

### 3.1 Characterization of PcCo/rGO hybrids

**Fig. 1:**
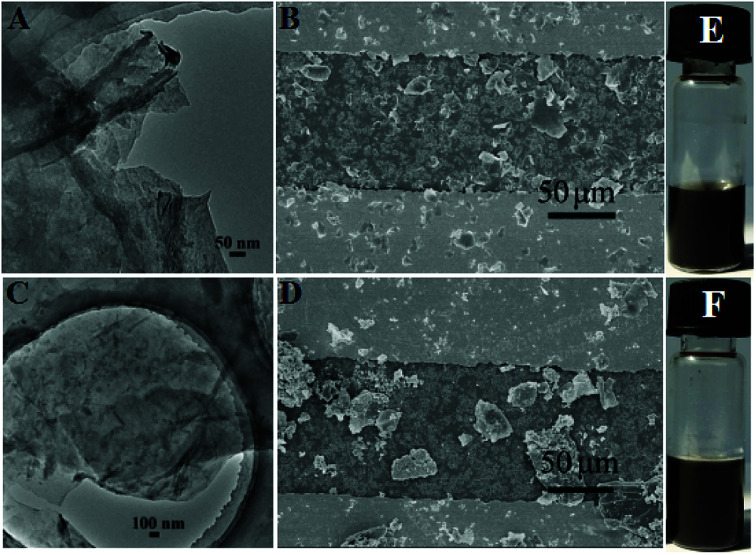
(A and C) TEM images and (B and D) SEM images of ABOPcCo/rGO and APcCo/rGO; and (E and F) optical photographs of the equivalent ABOPcCo/rGO and APcCo/rGO hybrids dispersed in DMF after sonicating at room temperature for 20 minutes.

The surface morphologies of the PcCo/rGO hybrids were characterized *via* TEM and SEM. The TEM images exhibit stacked few layered flakes with amorphous coatings (Fig. 1A and C), caused by the re-aggregation and folding of few layered graphene sheets and the functionalization of APcCo and ABOPcCo on the rGO surface.^35^ PcCo/rGO aqueous dispersions of 1.5 mg ml^−1^ (Fig. 1E and F) were drop cast onto the IDEs. The PcCo/rGO hybrids bridged gaps on the IDEs and provided the sensor with conductive and permeable channels for the diffusion of gas molecules (Fig. 1B and D). The SEM images also show that the ABOPcCo/rGO sheet sizes range from 20–400 μm^2^ and they are uniformly and loosely distributed between the two sides of the IDE; they are smaller and more even than those of APcCo/rGO. This agrees well with the surface morphologies of PcCo/rGO sensors at other concentrations of 0.5, 1.0 and 2.0 mg ml^−1^ (Fig. S2†), and can be attributed to the good dispersion of ABOPcCo/rGO hybrids evolving from ABOPcCo, containing four amine phenoxy groups, assembled on the surface of rGO. This is very useful in fabricating homogeneous gas sensors. Therefore, the influence of ABOPcCo and APcCo molecules on the dispersion of PcCo/rGO hybrids was further observed. Fig. S3A and B† show that the ABOPcCo/rGO and APcCo/rGO hybrids have good dispersibility in DMF after sonicating. Surprisingly, the ABOPcCo/rGO hybrid still exhibits excellent dispersibility, even after 2 months. The good dispersibility of the ABOPcCo/rGO dispersion bridges the IDE gap more effectively, playing important roles in the adsorption of gases and electron transport.^42,49,50^

**Fig. 2:**
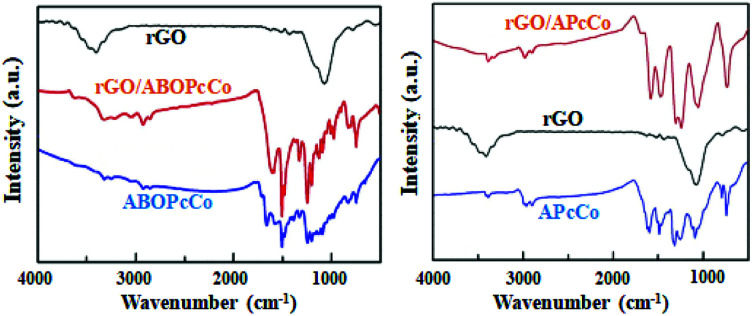
FT-IR spectra of rGO and the PcCo and PcCo/rGO hybrids.

**Fig. 3:**
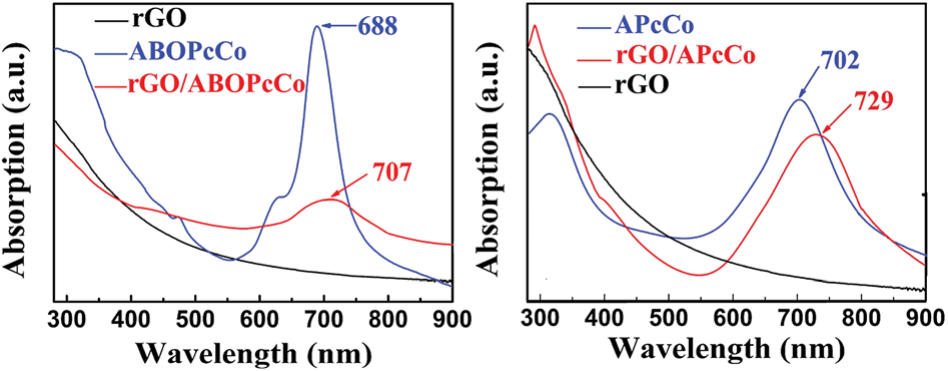
UV-vis spectra of rGO and the PcCo and PcCo/rGO hybrids.

**Fig. 4:**
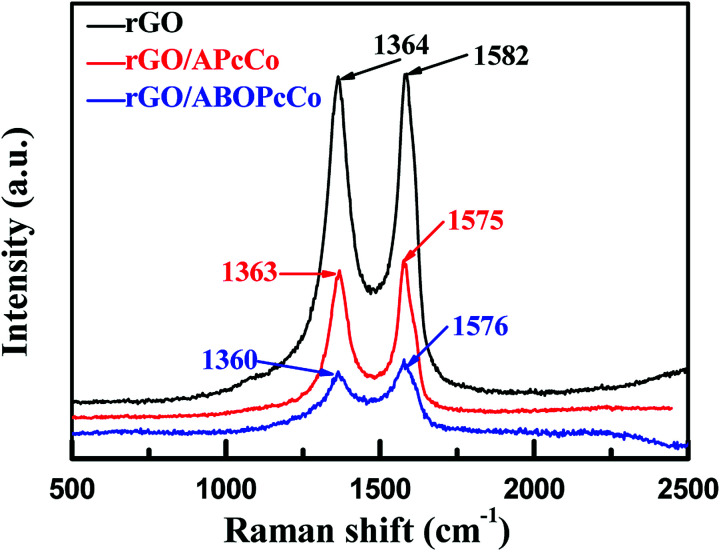
Raman spectra of rGO and the PcCo/rGO hybrids, obtained at *λ*_exc_ = 457.9 nm.

APcCo and ABOPcCo can be spontaneously loaded onto the surface of rGO *via* facile physical mixing, and the existence of PcCo/rGO hybrids is further confirmed *via* IR spectra, UV-vis spectra and Raman spectroscopy. FT-IR spectra of rGO, ABOPcCo and ABOPcCo/rGO hybrids are shown in Fig. 2A. The vibration peaks appearing at 1000–1650 cm^−1^, 3334 cm^−1^ and 3222 cm^−1^, and 2930 cm^−1^ and 2856 cm^−1^ are characteristic phthalocyanine ring, amino group N–H stretching and phenyl ring C–H stretching peaks in ABOPcCo, respectively.^51^ rGO shows its main characteristic peaks at 3419 cm^−1^ (*ν*_O–H_), 1634 cm^−1^ (*ν*_C–OH_) and 1080 cm^−1^ (*ν*_C–O–C_).^21^ The stretching vibration peaks at about 3300 cm^−1^, 2930 cm^−1^ and 1000–1650 cm^−1^ belong to the characteristic fingerprint absorption peaks of N–H, C–H and the Pc ring of ABOPcCo in the ABOPcCo/rGO hybrid. Moreover, the APcCo/rGO hybrid shows a similar phenomenon in Fig. 2B. These results indicate that PcCo/rGO hybrids have been obtained. The UV-vis spectra of rGO, PcCo and PcCo/rGO hybrids in DMF are shown in Fig. 3. The absorption peaks at 702 nm and 688 nm are the characteristic Q-band absorption peaks of APcCo and ABOPcCo. The Q-band absorption peak of ABOPcCo is about 14 nm lower than that of APcCo. This is due to the introduction of an aminophenoxy substituent, which reduces the electron donor power of the amino substituent and increases the gap between the highest occupied molecular orbital (HOMO) and lowest unoccupied molecular orbital (LUMO).^52^ These peaks are broadened and shifted to higher wavenumbers (by about 27 nm and 19 nm) in the APcCo/rGO and ABOPcCo/rGO hybrids. These results not only indicate that PcCo has been successfully loaded on rGO, but they also indicate that π–π stacking between PcCo and rGO exists in the hybrids.^52,53^ The Raman spectrum is used as a finger-print for rGO.^17^ As shown in Fig. 4, the strong peaks appearing at 1364 and 1582 cm^−1^ are the characteristic D and G bands of rGO, respectively. The D band is related to structural defects caused by oxidation or the attachment of functional groups. The G band is related to first-order scattering of E_2g_ symmetry.^21^ Compared with rGO, the Raman spectra of the ABOPcCo/rGO and APcCo/rGO hybrids are centered at 1360 and 1576 cm^−1^, and 1363 and 1575 cm^−1^, respectively, and are shifted to lower wavenumbers. Meanwhile, the *I*_D_/*I*_G_ values of the APcCo/rGO and ABOPcCo/rGO hybrids are 0.96 and 0.90, which are smaller than that of rGO (0.98). This indicates that the functionalization of APcCo and ABOPcCo results in less defective sites.^17^ These results confirm that APcCo and ABOPcCo are functionalized onto the rGO surface successfully *via* a noncovalent approach.

### 3.2 NH_3_ sensing properties of the PcCo/rGO hybrids

**Fig. 5:**
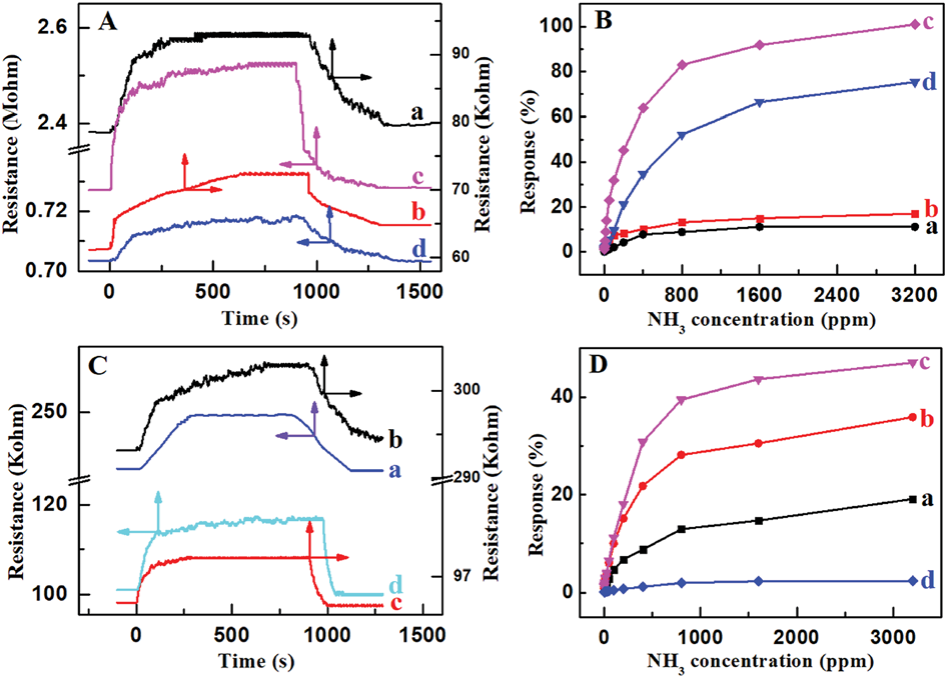
The resistance of (A) ABOPcCo/rGO and (C) APcCo/rGO hybrid sensors upon exposure to 50 ppm NH_3_ with PcCo/rGO aqueous dispersion concentrations of (a) 0.5, (b) 1.0, (c) 1.5, and (d) 2.0 mg ml^−1^, respectively, at 28 °C; and the relationship between the response of (B) ABOPcCo/rGO and (D) APcCo/rGO hybrid sensors and the concentration of NH_3_ with PcCo/rGO aqueous dispersion concentrations of (a) 0.5, (b) 1.0, (c) 1.5, and (d) 2.0 mg ml^−1^, respectively, at 28 °C.

The amount of PcCo/rGO hybrid bridging the IE gap and the resistance of the PcCo/rGO sensors can be controlled using *via* the concentrations of ABOPcCo/rGO and APcCo/rGO aqueous dispersions. Table S1† shows that the resistance values of the PcCo/rGO sensors are related to the concentrations of PcCo/rGO aqueous dispersions, as taken from Fig. 5A and C. For example, the ABOPcCo/rGO sensors show resistances of 2.38 ± 0.50, 0.71 ± 0.05, 0.07 ± 0.05 and 0.06 ± 0.05 MΩ with ABOPcCo/rGO aqueous dispersion concentrations of 0.5, 1.0, 1.5, and 2.0 mg ml^−1^, respectively (Fig. 5A). As has been shown, the resistance value of the PcCo/rGO sensor changes inversely to the concentration of PcCo/rGO aqueous dispersion. The higher the concentration of the ABOPcCo/rGO aqueous dispersion, the more ABOPcCo/rGO hybrid bridges the IDE gap, leading to reduced sensor resistance.^21^ More importantly, the NH_3_ sensing performance is related to the resistance values of the PcCo/rGO hybrids. It was found that the response increased first and then decreased as the resistance of the PcCo/rGO sensor decreased at 28 °C (Fig. 5B and D). The PcCo/rGO aqueous dispersion with a concentration of 1.5 mg ml^−1^ led to the preparation of a sensor showing excellent NH_3_ sensing performance. Therefore, this concentration was used to prepare sensors for gas sensing applications. The amount of PcCo/rGO hybrid bridging the IDE gap, and the number of absorption sites of PcCo/rGO for NH_3_ increase with an increase in the concentration of PcCo/rGO aqueous dispersion at low concentrations. However, a high concentration of PcCo/rGO reduces aqueous dispersion, leading to aggregation and reduced gas sensing performance.

As a control, the sensing responses of rGO and PcCo molecules towards NH_3_ were also studied. The response intensities are poor for the rGO and PcCo sensors (Fig. S4†). The rGO sensor shows poor recovery properties, and the resistance of the rGO sensor can't even recover its original resistance within an hour with an increase in the NH_3_ concentration. By contrast, the PcCo/rGO sensors exhibit remarkable response and recovery performances towards NH_3_, especially the ABOPcCo/rGO sensor. For example, the ABOPcCo/rGO sensor shows a response of 23.3% to 50 ppm NH_3_ gas (Fig. 5B), which is over 7 times and 15 times higher than the rGO and ABOPcCo sensors, respectively. The response and recovery times of the ABOPcCo/rGO and APcCo/rGO sensors to 50 ppm NH_3_ are 225 s and 225 s, and 80 s and 250 s, respectively, at 28 °C (Fig. S5†). Additionally, it is clear that the response of the ABOPcCo/rGO sensor is higher than that of the APcCo/rGO sensor (Fig. 5B and D). For example, the ABOPcCo/rGO sensor shows a 3.6 times higher response to 50 ppm NH_3_ gas than the APcCo/rGO sensor. The responses of the PcCo/rGO sensors to NH_3_ gas concentrations in the range of 3–50 ppm were approximately linear (Fig. S6†). For ABOPcCo/rGO, the slope of the linear fit was 0.421 ppm^−1^ and the correlation coefficient (*R*^2^) was 0.979. The limit of detection (LOD) was estimated to be 78 ppb at 28 °C (S/N = 3), according to eqn (2):2
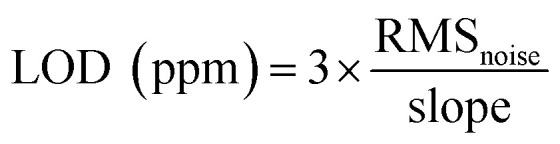
where RMS_noise_ is the standard deviation of the noise, which is calculated to be equal to 0.01 based on 11 data points from the baseline of the response curve.^54^ Similarly, the LOD of APcCo/rGO was estimated to be 1.3 ppm at 28 °C. In order to certify the effects of substituents on gas sensing properties, the gas sensing properties of PcNi/rGO were also studied. Fig. S7† shows the relationship between the responses of the PcNi/rGO hybrid sensors and the concentration of NH_3_. Similar to PcCo/rGO, the ABOPcNi/rGO sensor also shows a better response than the APcNi/rGO sensor.

**Fig. 6:**
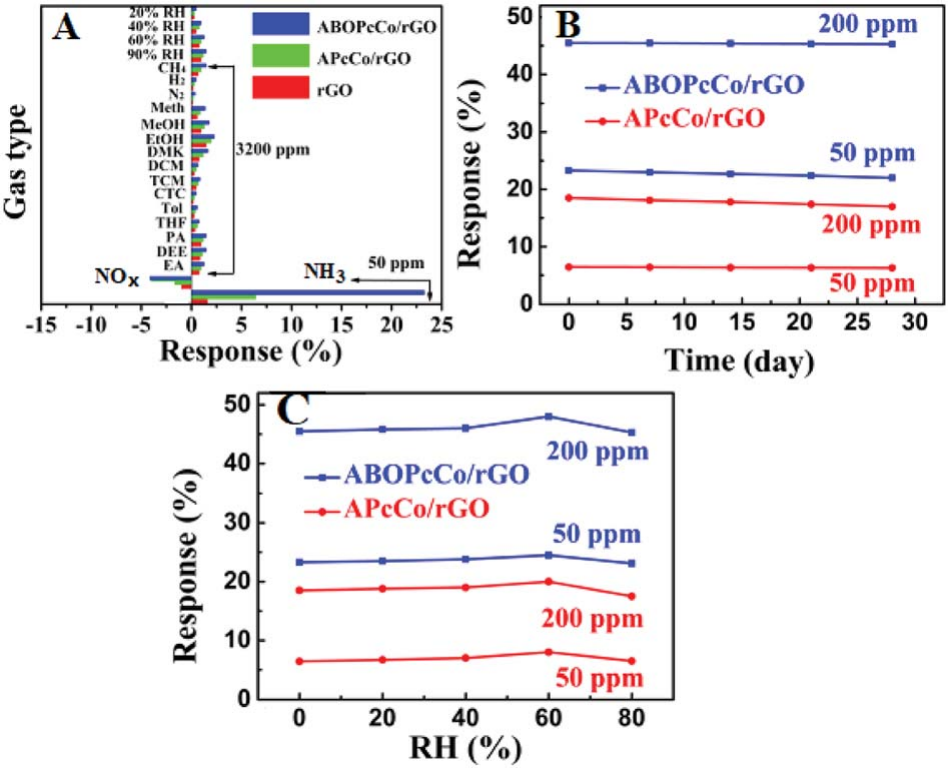
(A) Cross-sensitivities to various gases shown by the rGO and PcCo/rGO hybrid sensors: MeTH = methanal; MeOH = methanol; EtOH = ethanol; DMK = acetone; DCM = dichloromethane; TCM = trichloromethane; CTC = carbon tetrachloride; Tol = toluene; THF = tetrahydrofuran; PA = propionic acid; DEE = diethyl ether; and EA = ethyl acetate; (B) the responses of PcCo/rGO hybrid sensors to 50 and 200 ppm NH_3_ over long-term storage; and (C) the responses of PcCo/rGO hybrid sensors upon exposure to 50 and 200 ppm NH_3_ at different relative humidity levels at 28 °C.

Fig. 6A shows the selectivity of rGO, ABOPcCo/rGO and APcCo/rGO sensors towards several normal gas samples (NH_3_ and NO_*x*_: 50 ppm; other gases: 3200 ppm) and volatile organic compounds (VOCs: 3200 ppm) at different humidity levels at 28 °C. The responses of the rGO sensor to NH_3_, NO_*x*_, EtOH, MeOH, PA and 90% RH, other gases and VOCs are 3.2%, 2%, 1.5%, 1%, 1% and lower than 1%, respectively. Surprisingly, the responses of the ABOPcCo/rGO and APcCo/rGO sensors to NH_3_ increase to 23.3% and 6.4%, but the responses to other gases and different relative humidity levels are still less than 2%. It should be pointed out that the PcCo/rGO sensors exhibit high responses to NH_3_ and low responses to NO_*x*_ at the same concentration (50 ppm), which is usually not easily achieved. The results demonstrate that the functionalization of APcCo and ABOPcCo on rGO offers a very effective way to tune and boost the gas sensing performance, such as producing improved responses and selectivities.

Fig. 6B shows the long-term stabilities of the ABOPcCo/rGO and APcCo/rGO sensors upon exposure to 200 ppm and 50 ppm NH_3_ over 30 days at 28 °C. The responses of the ABOPcCo/rGO and APcCo/rGO sensors up to 30 days reduced by less than 5%, indicating that the PcCo/rGO sensors display excellent long-term stability. Meanwhile, the ABOPcCo/rGO sensor also displays 3.6 and 2.4 times higher responses to 50 ppm and 200 ppm NH_3_, respectively, than the APcCo/rGO sensor. Moreover, the effects of humidity on the NH_3_-sensing properties of the PcCo/rGO sensors were also studied. Fig. 6C shows the responses of the ABOPcCo/rGO and APcCo/rGO hybrid sensors upon exposure to 200 ppm and 50 ppm NH_3_ at different relative humidity levels at 28 °C. As can be seen, the responses of the PcCo/rGO sensors did not change obviously below 60% RH. However, the response decreases under a high RH of 80%, which might be ascribed to competitive adsorption between NH_3_ and H_2_O molecules.^55,56^

**Fig. 7:**
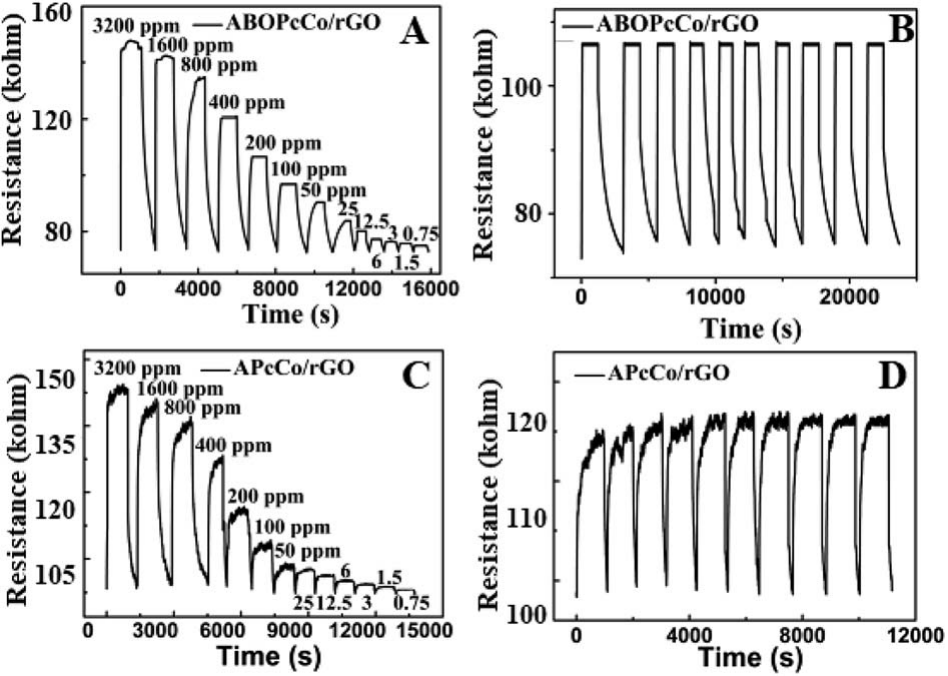
The resistance of (A) ABOPcCo/rGO and (C) APcCo-rGO hybrid sensors upon exposure to varying concentrations of NH_3_ with a PcCo/rGO aqueous dispersion concentration of 1.5 mg ml^−1^; and ten sensing cycles of (B) ABOPcCo/rGO and (D) APcCo-rGO hybrid sensors being exposed to 200 ppm NH_3_ with a PcCo/rGO aqueous dispersion concentration of 1.5 mg ml^−1^ at 28 °C.

Fig. 7 displays the dynamic resistance curves of PcCo/rGO sensors upon exposure to different NH_3_ concentrations, from 3200 ppm to 750 ppb, and in 200 ppm NH_3_ gas over ten continuous cycles at 28 °C. The resistance goes through a rapid increase followed by a slow increase in NH_3_ gas. Then, the resistance sharply decreases when the sensors are exposed to air again (Fig. 7A and C). The sensors can detect relatively low concentrations of NH_3_, as low as 78 ppb. Fig. 7B and D show dynamic resistance curves over ten cycles of exposing the ABOPcCo/rGO and APcCo/rGO sensors to 200 ppm NH_3_. The resistance curves show similar continuous recycling and the error is less than 3% over ten continuous cycles. The sensor shows a fast recovery time. Clearly, the PcCo/rGO sensors have good stability and repeatability, which are crucial for further applications. The high sensitivities, low detection limits and fast response and recovery times of our NH_3_ sensors are also significantly superior to previously reported sensors (as shown in Table S2†).

To obtain further insight into the effects of amino substituents on gas sensing properties, a substituent-free phthalocyanine cobalt/rGO (FPcCo/rGO) hybrid was synthesized *via* noncovalent interactions. The dynamic response, reproducibility, and relationship between the response of the PcCo/rGO sensor and the concentration of NH_3_ were also investigated (as shown in Fig. S8†). It's clear that the response of the FPcCo/rGO sensor increases as the concentration of NH_3_ is increased. Although the FPcCo/rGO sensor exhibits fast response and recovery times and good reproducibility, its response is so much lower than those of the ABOPcCo/rGO and APcCo/rGO sensors that no response can be observed at concentrations less than 12.5 ppm NH_3_. As shown in Fig. S8D,† the responses of the various sensors decrease in the order: ABOPcCo/rGO > APcCo/rGO > FPcCo/rGO > rGO. Owing to the same central metal phthalocyanine loading, the discrepancies in responses are mainly due to differences between the PcCo compounds or, to be more specific, the substituents, for example, the electron donating ability and the electron cloud density, especially interaction effects between NH_3_ and different PcCo compounds. Amino groups have stronger electron-donating capabilities. They can provide more electrons to combine with the holes in the p-type semiconductors of PcCo and rGO. The combination of electrons and holes can reduce charge transfer between APcCo/rGO and the adsorbed gas molecules, reducing NH_3_ gas and leading to further speeding up the recovery time. On the contrary, aminophenoxy groups have weaker electron-donating capabilities than amino groups. This can improve charge transfer between ABOPcCo/rGO and the adsorbed gas molecules, reducing NH_3_ gas and leading to a further increase in the gas response.^38^ These results further indicate that the substituents of PcCo molecules are of primary importance in tuning the gas sensing performance. The effects of amino substituents on the sensing properties will be discussed using DFT studies.

### 3.3 The NH_3_ sensing mechanism of PcCo/rGO hybrids

The NH_3_ sensing mechanism of the PcCo/rGO hybrids is based on the resistance changes of the PcCo/rGO sensors upon exposure to NH_3_, which are attributed to charge transfer between PcCo/rGO and adsorbed gas molecules. A reducing gas (NH_3_) donates electrons to PcCo/rGO, which could lead to a decrease in charge carriers (holes) and an increase in the electrical resistance of the hybrid (Fig. 5 and 7). This hypothesis is further confirmed upon interaction with an oxidizing gas, such as NO_2_. An oxidizing gas (NO_2_) withdraws electrons from PcCo/rGO, which could lead to a contrary change in the electrical resistance of a hybrid (Fig. 6A). ABOPcCo and APcCo loaded rGO hybrids display improved NH_3_ sensing performances, which are attributed to the following factors. Firstly, rGO as a conductive agent has a large specific surface area and high carrier mobility and plays an important role in the electronic properties of PcCo/rGO hybrids, which show great promise for sensor performance enhancement. Secondly, the functionalization of APcCo and ABOPcCo on the surface of rGO offers more active NH_3_ sensing sites. Meanwhile, the amino groups of APcCo, as a stronger electron-donating group, provide more electrons to combine with the holes in the p-type semiconductors of APcCo and rGO, weakening charge transfer between APcCo/rGO and adsorbed gas molecules, reducing NH_3_ gas and accelerating the recovery time. On the contrary, the aminophenoxy groups of ABOPcCo weaken the donation power, decreasing the combination of electrons and holes and increasing charge transfer between ABOPcCo/rGO and adsorbed gas molecules; this reduces NH_3_ gas and enhances the NH_3_ sensing response of ABOPcCo/rGO for NH_3_ molecules, decreasing the recovery ability. These results show promise in tuning the sensitivity and recovery performance of rGO-based gas sensors, which was confirmed *via* DFT calculations. Due to less interaction between NH_3_ and rGO, only the interactions between NH_3_ and PcCo molecules were calculated. Table 1 shows the net charge on the NH_3_ moiety and the distance between cobalt and NH_3_. Fig. S9† shows the structures of adsorbed NH_3_–PcCo molecules, with top and side views. The smaller the distance between cobalt and NH_3_, the larger the net charge on NH_3_, the greater the charge transfer between PcCo and NH_3_ gas, and the better the gas sensing performance of PcCo/rGO for NH_3_. The distance in ABOPcCo–NH_3_ is shorter, and the net charge on the NH_3_ moiety is larger than that for APcCo–NH_3_ and FPcCo–NH_3_, so the NH_3_ sensing properties of ABOPcCo/rGO are better than those of APcCo/rGO and FPcCo/rGO. Thirdly, charge transfer between PcCo and rGO can be achieved *via* π–π stacking interactions, which can accelerate charge transfer between the PcCo/rGO hybrids and adsorbed NH_3_ molecules. In order to verify the electron transportation abilities, electrochemical impedance spectroscopy (EIS) data of PcCo and PcCo/rGO hybrids were studied (Fig. S10†). The semicircle in the high-frequency range for PcCo/rGO is smaller than that of PcCo, which corresponds to the charge transfer resistance (*R*_ct_ in Table S3†). *R*_ct_ is related to electron transportation in the gas sensor. The lower the value of *R*_ct_, the stronger the electron transport. The *R*_ct_ values of the PcCo/rGO hybrids are lower than that of PcCo. The electrons can easily transfer from NH_3_ to PcCo/rGO. Moreover, the *R*_ct_ value of ABOPcCo/rGO is much lower than that of APcCo/rGO. Therefore, the ABOPcCo/rGO sensor displays better NH_3_ sensing performance.

**Table 1:** Calculated adsorption energies, major bond distances, and net charges in the adsorption of NH_3_ on the PcCo examples

Sub.^a^	Net charge^b^	*d*(M–R)^c^ (Å)
ABOPcCo	0.168	2.214
APcCo	0.162	2.216
PcCo	0.160	2.240

a Substrate.

b The net charge on NH_3_.

c The distance between the metal and NH_3_.

## 4. Conclusions

Three NH_3_ gas sensors based on ABOPcCo/rGO, APcCo/rGO and FPcCo/rGO hybrids have been fabricated with significantly improved sensitivities, selectivities and recoveries through optimizing the concentration of a PcCo/rGO aqueous dispersion. For example, compared with pure rGO, ABOPcCo/rGO, APcCo/rGO and FPcCo/rGO hybrids showed twenty-three-fold, six-fold and two-fold higher responses to 50 ppm NH_3_, respectively, and improved recovery times for NH_3_ sensing. Additionally, the performances of the PcCo/rGO hybrids may be tuned by adjusting the PcCo substituent. Compared with many existing NH_3_ sensors, the obtained sensors, especially the ABOPcCo/rGO sensor (23.3% to 50 ppm NH_3_, with a LOD of 78 ppb and a 250 s recovery time), exhibit better responses. These performances are mainly ascribed to: (a) the aminophenoxy groups reducing the electron donation power of the amino groups and increasing the charge transfer between PcCo/rGO and NH_3_; (b) rGO providing high conductivity and continuous pathways for charge transportation; and (c) PcCo/rGO hybrids showing excellent electron transportation abilities. EIS and DFT calculations confirm that the aminophenoxy groups of PcCo play a critical role in the NH_3_ sensing performance. This systematic study provides a valid way to improve the performances of rGO based NH_3_ sensors.

## Conflicts of interest

There are no conflicts to declare.

## Supplementary Material

RA-008-C8RA07509C-s001

## References

[cit1] Lee S. W., Lee W., Hong Y., Lee G., Yoon D. S. (2018). Sens. Actuators, B.

[cit2] Zhang J., Qin Z. Y., Zeng D., Xie C. (2017). Phys. Chem. Chem. Phys..

[cit3] Tanguy N. R., Thompson M., Yan N. (2018). Sens. Actuators, B.

[cit4] Varghese S. S., Lonkar S., Singh K. K., Swaminathan S., Abdala A. (2015). Sens. Actuators, B.

[cit5] kaushik A., Kumar R., Arya S. K., Nair M., Malhotra B. D., Bhansali S. (2015). Chem. Rev..

[cit6] Korotcenkov G., Cho B. K. (2017). Sens. Actuators, B.

[cit7] Cheng J. P., Wang J., Li Q. Q., Liu H. G., Li Y. (2016). J. Ind. Eng. Chem..

[cit8] Zhu L., Zeng W. (2017). Sens. Actuators, A.

[cit9] Mao S., Lu G. H., Chen J. H. (2014). J. Mater. Chem. A.

[cit10] Yuan W. J., Shi G. Q. (2013). J. Mater. Chem. A.

[cit11] Georgakilas V., Otyepka M., Bourlinos A. B., Chandra V., Kim N., Kemp K. C., Hobza P., Zboril R., Kim K. S. (2012). Chem. Rev..

[cit12] Perreault F., Fonseca de Faria A., Elimelech M. (2015). Chem. Soc. Rev..

[cit13] Llobet E. (2013). Sens. Actuators, B.

[cit14] Wei J. W., Liang B., Cao Q. P., Mo C. T., Zheng Y. M., Ye X. S. (2017). RSC Adv..

[cit15] Khurshid F., Jeyavelan M., Takahashi K., Sterlin Leo Hudson M., Nagarajan S. (2018). RSC Adv..

[cit16] Schedin F., Geim A. K., Morozov S. V., Hill E. W., Blake P., Katsnelson M. I., Novoselov K. S. (2007). Nat. Mater..

[cit17] Wang X., Li X., Zhao Y., Chen Y., Yu J., Wang J. (2016). RSC Adv..

[cit18] Wei X. L., Chen Y. P., Liu W. L., Zhong J. X. (2012). Phys. Lett. A.

[cit19] Salehi-Khojin A., Estrada D., Lin K. Y., Bae M. H., Xiong F., Pop E., Masel R. I. (2012). Adv. Mater..

[cit20] Zhao H. Y., Fan S. Q., Chen Y., Feng Z. H., Zhang H., Pang W., Zhang D. H., Zhang M. L. (2017). ACS Appl. Mater. Interfaces.

[cit21] Wu J., Tao K., Zhang J., Guo Y. Y., Miao J. M., Norford L. K. (2016). J. Mater. Chem. A.

[cit22] Yavari F., Koratka N. (2012). J. Phys. Chem. Lett..

[cit23] Zhang S., Zhang D., Sysoev V. I., Sedelnikova O. V., Asanov I. P., Katkov M. V., Song H. H., Okotrub A. V., Bulusheva L. G., Chen X. H. (2014). RSC Adv..

[cit24] Yang Y., Sun L., Dong X. T., Yu H., Wang T. T., Wang J. X., Wang R. H., Yu W. S., Liu G. X. (2016). RSC Adv..

[cit25] Karaduman I., Er E., Çelikkan H., Erk N., Acar S. (2017). J. Alloys Compd..

[cit26] Triet N. M., Duy L. T., Hwang B., Hanif A., Siddiqui S., Park K., Cho C. Y., Lee N. E. (2017). ACS Appl. Mater. Interfaces.

[cit27] Huang X. L., Hu N. T., Gao R. G., Yu Y., Wang Y. Y., Yang Z. (2012). et al.. J. Mater. Chem..

[cit28] Yang Y. J., Yang X. J., Yang W. Y., Li S. B., Xu J. H., Jiang Y. D. (2014). RSC Adv..

[cit29] Huang X. L. , Hu N. T., Wang Y. Y. and Zhang Y. F., Leading Edge of Micro-Nano Science and Technology, 2013, vol. 669, p. 79

[cit30] Ye Z. B., Jiang Y. D., Tai H. L., Yuan Z. (2014). Integr. Ferroelectr..

[cit31] Kumar A., Joshi N., Samanta S., Singh A., Debnath A. K., Chauhan A. K., Roy M., Prasad R., Roy K., Chehimi M. M., Aswal D. K., Gupta S. K. (2015). Sens. Actuators, B.

[cit32] Kumar A., Brunet J., Varenne C., Ndiaye A., Pauly A., Penza M., Alvisi M. (2015). Sens. Actuators, B.

[cit33] Sizun T., Bouvetn M., Suisse J. (2012). Talanta.

[cit34] Wang Y. Y., Hu N. T., Zhou Z. H., Xu D., Wang Z., Yang Z., Wei H., Kong E. S. W., Zhang Y. F. (2011). J. Mater. Chem..

[cit35] Ragoussi M., Malig J., Katsukis G., Butz B., Spiecker E., Torre G., Torres T., Guldi D. M. (2012). Angew. Chem., Int. Ed..

[cit36] Zhu J. H., Li Y. X., Chen Y., Wang J., Zhang B., Zhang J. J., Blau W. J. (2011). Carbon.

[cit37] Karousis N., Ortiz J., Ohkubo K., Hasobe T., Fukuzumi S., Sastre-Santos Á., Tagmatarchis N. (2012). J. Phys. Chem. C.

[cit38] Wu H., Chen Z. M., Zhang J. L., Wu F., He C. Y., Wang B., Wu Y. Q., Ren Z. Y. (2016). J. Mater. Chem. A.

[cit39] D'Souza F., Ito O. (2009). Chem. Commun..

[cit40] Zhou X. Q., Wang X. L., Wang B., Chen Z. M., He C. Y., Wu Y. Q. (2014). Sens. Actuators, B.

[cit41] Li Y., Wang B., Yu Z. Y., Zhou X. Q., Kang D., Wu Y. Q., Chen Z. M., He C. Y., Zhou X. (2017). RSC Adv..

[cit42] Wang B., Wu Y. Q., Wang X. L., Chen Z. M., He C. Y. (2014). Sens. Actuators, B.

[cit43] Ma X. F., Sun J. Z., Wang M., Hu M., Li G., Chen H. Z., Huang J. (2006). Sens. Actuators, B.

[cit44] Parkhomenko R. G., Sukhikh A. S., Klyamer D. D., Krasnov P. O., Gromilov S., Kadem B., Hassan A. K., Basova T. V. (2017). J. Phys. Chem. C.

[cit45] Yu Z. Y., Wang B., Li Y., Kang D., Chen Z. M., Wu Y. Q. (2017). RSC Adv..

[cit46] Yanaia T., Tew D. P., Handyb N. C. (2004). Chem. Phys. Lett..

[cit47] Glendening E. D. , Carpenter A. E. and Weinhold F., NBO, version 3.1, 1995

[cit48] Frisch M. J. , et al., Gaussian 09, Revision B01, Gaussian, Inc., Wallingford, CT, 2009

[cit49] Li P., Ding Y., Wang A., Zhou L., Wei S. H., Zhou Y. M., Tang Y. W., Chen Y., Cai C. X., Lu T. H. (2013). ACS Appl. Mater. Interfaces.

[cit50] Li P., Liu H. L., Yang J., Sun D. M., Chen Y., Zhou Y. M., Cai C. X., Lu T. H. (2013). J. Mater. Chem. B.

[cit51] Ogbodu R. O., Limson J. L., Prinsloo E., Nyokong T. (2015). Synth. Met..

[cit52] Mani V., Devasenathipathy R., Chen S. M., Gu J. A., Huang S. T. (2015). Renewable Energy.

[cit53] Hou K., Huang L., Qi Y. B., Huang C. X., Pan H. B., Du M. (2015). Mater. Sci. Eng., C.

[cit54] Song L., Wei Z. R., Wang B. C., Luo Z., Xu S. M., Zhang W. K., Yu H. X., Li M., Huang Z., Zang J. F., Yi F., Liu H. (2016). Chem. Mater..

[cit55] Liu C. H., Tai H. L., Zhang P., Ye Z. B., Su Y. J., Jiang Y. D. (2017). Sens. Actuators, B.

[cit56] Song H., Li X., Cui P., Guo S. X., Liu W. H., Wang X. L. (2017). Sens. Actuators, B.

