# Supplementary material for: The effects of amino substituents on the enhanced ammonia sensing performance of PcCo/rGO hybrids
Source: RSC Adv. 2018 Dec 11;8(72):41280–7. doi: 10.1039/c8ra07509c (PMC9091620; doi:10.1039/c8ra07509c)
Supplement: RA-008-C8RA07509C-s001 [file RA-008-C8RA07509C-s001.pdf]

Electronic Supplementary Material (ESI) for RSC Advances.
This journal is © The Royal Society of Chemistry 2018

The effects of amino substituent on enhanced ammonia sensing performance of PcCo/rGO hybrids†

Bin Wang,[*a] Xiaolin Wang,[*b] Xiaocheng Li,[a] Zhijiang Guo,[a] and Xin Zhou,[c] Yiqun Wu,[ad]

[a] Key Laboratory of Functional Inorganic Material Chemistr, Ministry of Education, School of Chemistry and Materials Science, Heilongjiang University, Harbin 150080, P. R. China. E-mail: wangbin@hlju.edu.cn.

[b] School of Material and Chemical Engineering, Heilongjiang Institute of Technology, Harbin 150050, P. R. China

[c] MIIT Key Laboratory of Critical Materials Technology for New Energy Conversion and Storage, School of Chemistry and Chemical Engineering, Harbin Institute of Technology, Harbin, P. R. China

[d] Shanghai Institute of Optics and Fine Mechanics, Chinese Academy of Sciences, Shanghai 201800, P. R. China.

Tel.: +86 451 86609121

Fax: +86 451 86673647

1. Experimental detail

1.1 Materials

All chemicals were analytical grade and commercially available and used without further purification. 3-nitrophthalonitrile was purchased from Sigma-Aldrich Co . LLC., and was used without further purification. The synthesis scheme of Tetra-α-(p-aminobenzyloxy) phthalocyanine cobalt (ABOPcCo) and Tetra-α-aminophthalocyanines cobalt (APcCo) is shown in scheme S1.

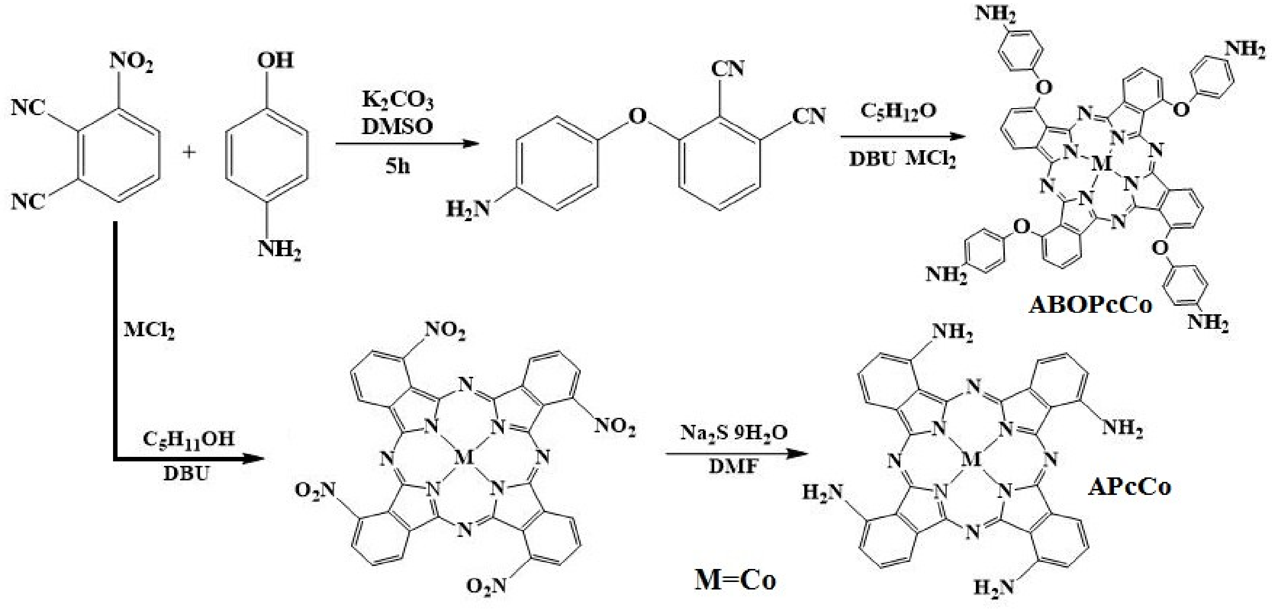

Scheme S1. Synthesis scheme of ABOPcCo and APcCo.

1.2 Characterization

EI and MALDI-TOF mass spectra were performed using an Agilent spectrometer (HP 5973N) and a Bruker microflex LT (Bruker Daltonics, Bremen, Germany) mass spectrometer, respectively. Scanning electron microscopy (SEM) images were recorded with a Hitachi S-4800 field emission scanning electron microscope operating at 15 kV. Samples were drop-deposited onto the interdigitated electrodes and measured directly. UV/Vis absorption spectra were recorded with an UV-2700 spectrometer (SHIMADZU, Jap). FT-IR spectra were recorded on a Spectrum two spectrometer (PerkinElmer).

1.3 Synthesis of 3- aminophenoxyphthalonitrile

3-nitrophthalonitrile (1.73 g, 0.02 mol) was dissolved in Dimethyl sulphoxide DMSO (20 mL) under a nitrogen atmosphere and p-aminophenol (3.27 g, 0.03 mol) was added to the solution. After stirring for 10 min, finely ground anhydrous potassium carbonate (K_2CO_3) (1.67 g, 0.012 mol) was added batch-wise over 5 h with efficient stirring at room temperature. Then the solution was poured

into ice-water and then was filtrated. The filter cake was poured several times with boiling water until the filtrate was clear. After the crude product was recrystallized from methanol. EI-MS Calcd (Found): m/z= 235.11 (235.24).

1.4 Synthesis of ABOPcCo

3- aminophenoxyphthalonitrile (1.0 g, 4.2 mmol) was dissolved in 10 ml of anhydrous n-pentanol and anhydrous cobalt dichloride (1.2 mmol) and 0.3 ml of 1,8-dicyanobicycio- [5.4.0]-undec-7-ene (DBU) were added to this solution. The solution was refluxed for 12 h with continuous stirring under a nitrogen atmosphere. The reaction mixture was cooled and product was precipitated in methanol solution. The precipitate was suction filtered and washed several times with methanol until the filtrate was clear. The crude product was extracted with acetone and water by Soxhlet extraction method. The product was dried in a vacuum oven at 50 ℃ to obtain ABOPcCo.

ABOPcCo: Electronic absorption spectrum (UV-Vis) in DMF: max (nm) = 686. FT-IR spectra (KBr pellets) ν:1612, 1494, 1246, 813, 739 cm^{-1}. MALDI-TOF-MS (Fig S1A) Calcd (found): m/z = 1000.82 (999.90) [M^+].

1.5 Synthesis of APcCo

3-Niyrophthalonitrile (1.0g, 4.7 mmol) was dissolved in 10 ml of anhydrous n-pentanol with anhydrous cobalt dichloride (1.2 mmol) and 0.4 ml of 1,8-dicyanobicycio- [5.4.0]-undec-7-ene (DBU). The solution was refluxed for 6h with continuous stirring under a N_2 atmosphere. The reaction mixture was cooled and product was precipitated in methanol solution. The precipitate was suction filtered and washed several times with the dilute hydrochloric acid and sodium hydroxide, and then washed with distilled water until the pH of the supernatant approached 7. The rough product was washed several times with alcohol and then dried in a vacuum oven at 50 ºC to obtained tetra-α-nitrophthalocyanine metal. Then, tetra-α-nitrophthalocyanine metal was dissolved in DMF with Sodium sulfide. The solution was heated up to 60℃ for 2h. The precipitate was washed with ultrapure water until the pH of the supernatant approached 7, and then centrifugal washed with methanol until the centrifugal supernatant is colorless. The product was dried in a vacuum oven at 50 ºC to obtain APcCo.

APcCo: Electronic absorption spectrum (UV-Vis) in DMF: max (nm) = 701, 639. FT-IR spectra (KBr pellets) ν: 1687, 1592, 1489, 1325, 1265, 1119, 1093, 800, 739 cm^{-1}. MALDI-TOF-MS (Fig S1B) Calcd (found): m/z = 631.11 (631.51) [M^+].

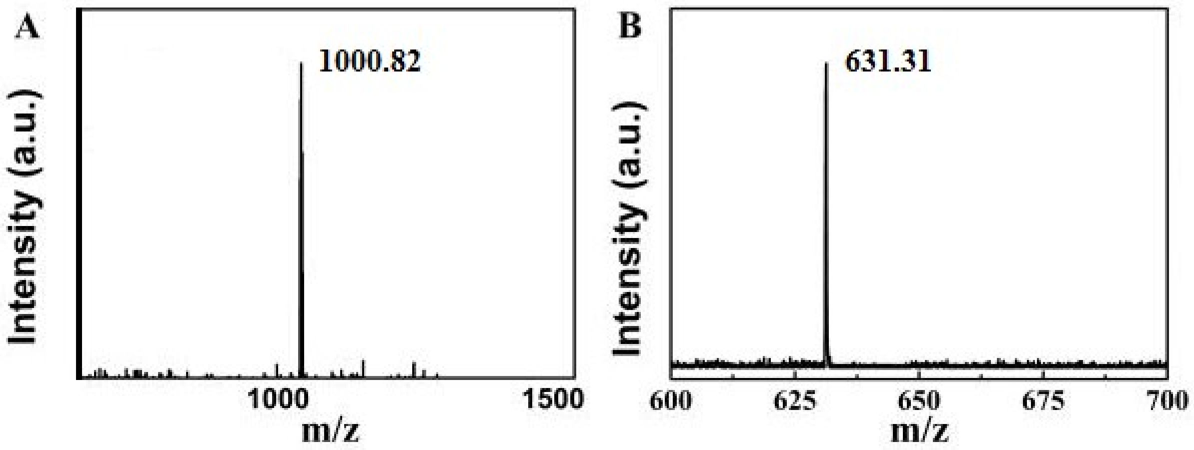

Fig. S1 MALDI-TOF mass spectra of (A) ABOPcCo and (B) APcCo.

1.6 Synthesis of FPcCo

The FPcCo complex was synthesized by a procedure similar to that of the ABOPcCo and APcCo complex, except that 1,2-benzendikarbonitril was used. The product was isolated to give green powders. Electronic absorption spectrum (UV-Vis) in DMF: max (nm) = 655, FT-IR spectra (KBr pellets) ν: 1522, 1416, 1325, 1112, 731 cm^{-1}. MALDI-TOF-MS Calcd (found): m/z = 571.46 (572.48) $[M^{+}]$.

Result and discussion

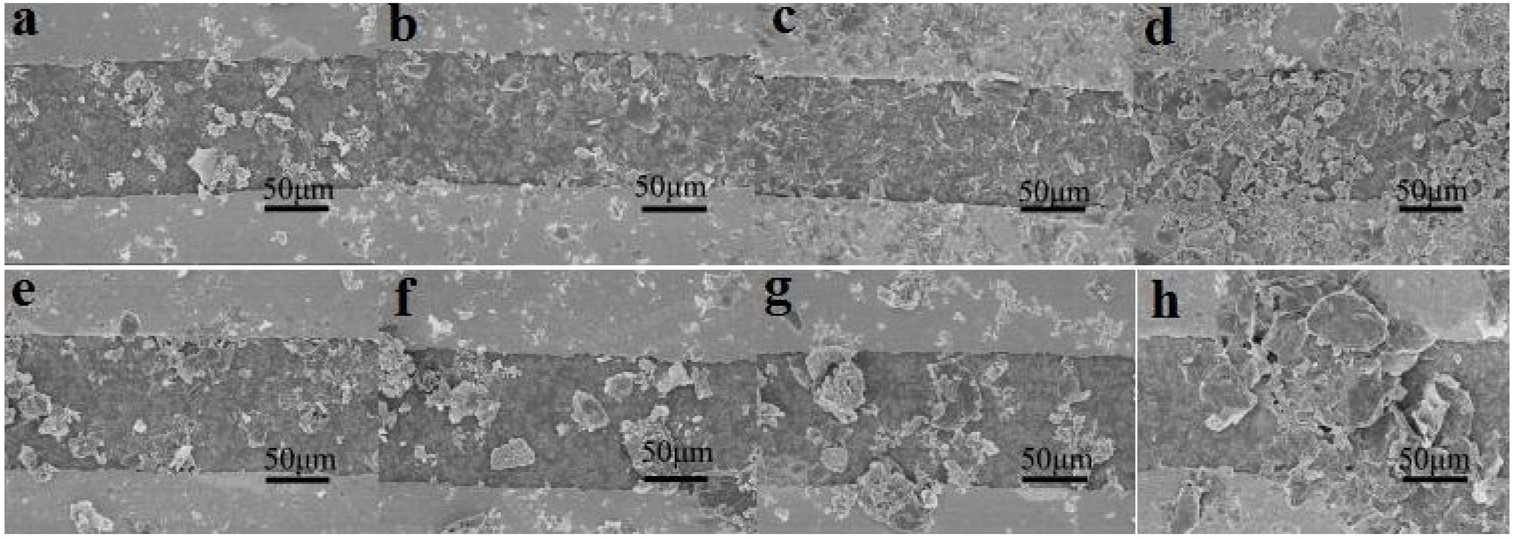

Fig. S2 SEM images of (a-d) ABOPcCo/rGO and (e-f) APcCo/rGO; the aqueous dispersion concentrations of (a,e) 0.5, (b,f) 1.0, (c,g) 1.5, and (d,h) 2.0 mg ml^{-1}, respectively.

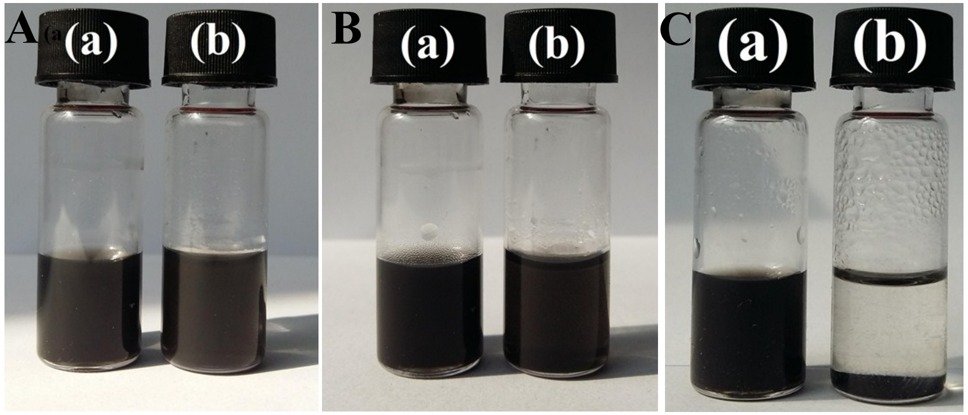

Fig. S3 Optical photographs of the equivalent (a) ABOPcCo/rGO and (b) APcCo/rGO. hybrids dispersed in DMF after sonicating at room temperature for 30 minutes (A), and after standing at room temperature for 5 days (B) and 60 days (C).

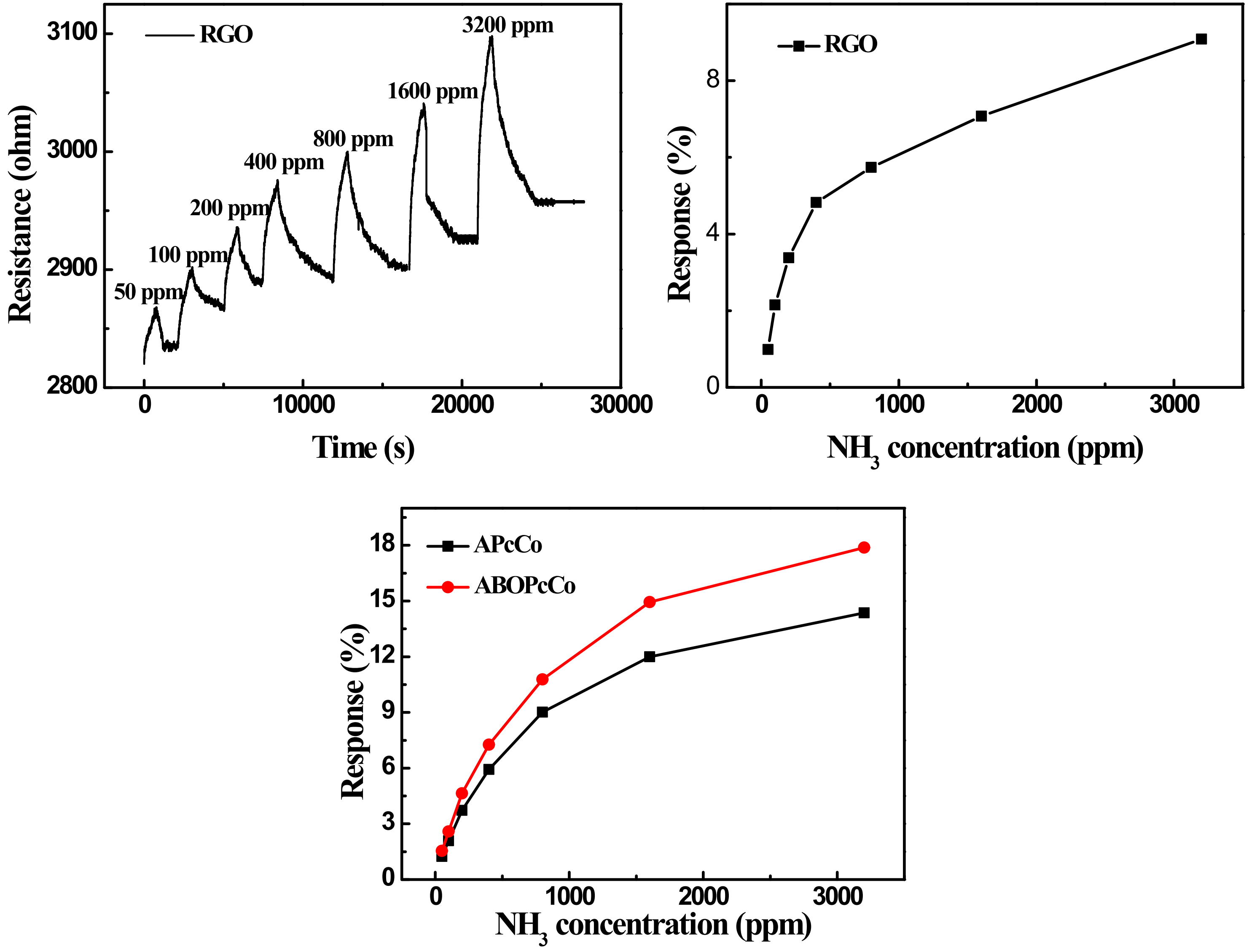

Fig. S4 (A) Resistance of rGO sensor upon exposure to varying concentrations of NH_3; relationship of the response of (B) rGO and (C) PcCo sensors to the concentration NH_3.

Table S1 Resistance value of PcCo/rGO sensors relate to the concentration of PcCo/rGO aqueous dispersion.

Sub<br>Resistance (Mohm)<br>Conc (mg/l)	0.5	1.0	1.5	2.0
ABOPcCo/rGO	2.38	0.71	0.07	0.06
APcCo/rGO	0.23	0.24	0.10	0.09

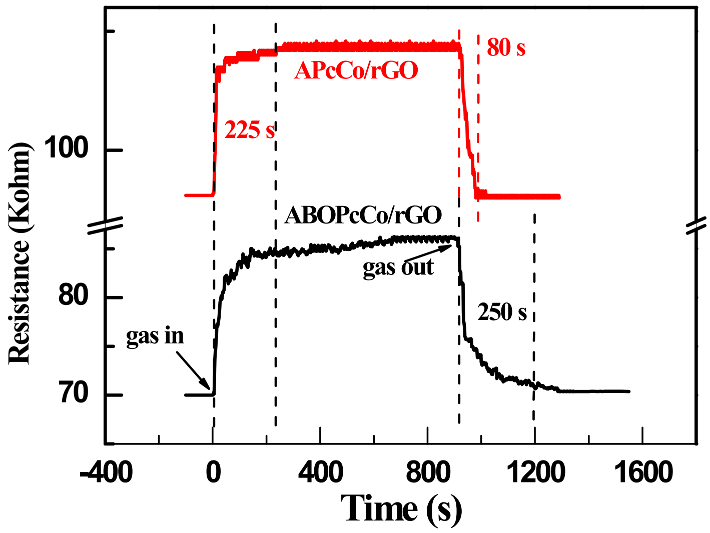

Fig. S5 Resistance of (A) ABOPcCo/rGO and (C) APcCo/rGO hybrid sensors upon exposure to 50 ppm NH_3.

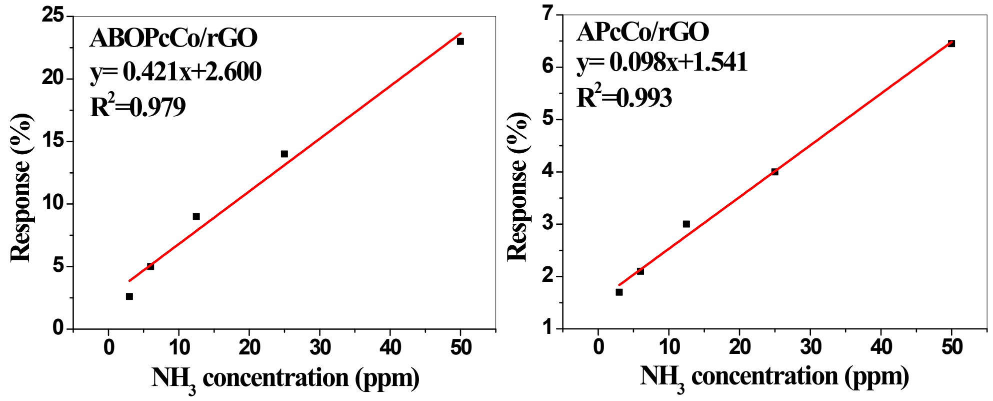

Fig. S6 Relationship of the response of ABOPcCo/rGO and APcCo/rGO hybrid sensors to the concentration NH_3 with the PcCo/rGO aqueous dispersion concentrations of 1.5 mg ml^{-1} at 28°C.

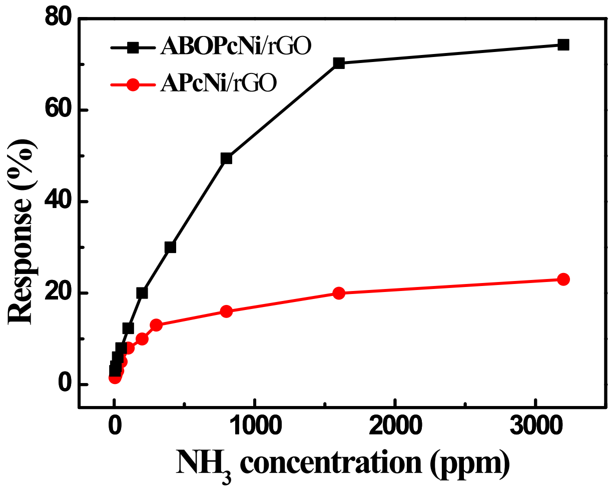

Fig. S7 Relationship of the response of PcNi/rGO hybrid sensors to the concentration NH_3.

Table S2. Comparison of the detection performances of different NH_3 sensors

Sensor material	Response(%)/Detection conc. (ppm) [b]	Detection limit(ppm)[a]	Working temperature (°C)[c]	Response time(s)/Detection conc.(ppm)[b]	Recovery time (s)/Detection conc.(ppm)[b]	Detection range (ppm)	Ref.
SnO_2-WO_3 nanofilm	7.1 (250)	1.7	300	—	—	50-1000	1
Pt-loaded WO_3	13.6 (200)	50	125	43 (200)	273 (200)	50-1500	2
CuO nanowires	3.1/100	10	200	360/100	1800/100	10-100	3
ZnO–PANI	110 (100)	10	RT	21 (100)	61 (100)	20-100	4
CeO_2@PANI	6.5/50	2	RT	57.6/50	300/50	2-400	5
PANI/Ag nanotubes	3/100	5	RT	~3000/100	~3000/100	5-100	6
CNT-on-paper	~2.8/100	~5	RT	60/100	>4/100(not fully recover)	10-100	7
Au-coated CNT yarn	~2.0/50	0.5	RT	>300/50	2700/50	0.5-500	8
PANi@CNT film	—	50	RT	~1200/50	—	50-5000	9
rGO	5.5/200	200	RT	900	>1200/200(not fully	200-2800	10

					recover)		
Graphene	100/1000	0.5	RT	3600	3600	—	11
Graphene-SnO	3.5 (5)	5	RT	15 (200)	30 (200)	5-200	12
ABOPcCo/rGO	23.3/50	0.078	RT	225/50	250/50	0.75-3200	This
APcCo/rGO	6.4/50	1.3	RT	225/50	80/50	0.75-3200	work[d]

[a] If the sensor detection limit was not explicitly provided in the original report, then the lowest tested analyte concentration is listed.

[b] If the response (%), response time (s) or recovery time (s) of the sensor was not explicitly provided in the original report, then the estimate from the curve in that report is listed.

[c] RT, abbreviation for room temperature.

[d] sensor prepared with the PcCo/rGO aqueous dispersion concentrations of 1.5 mg ml^{-1}

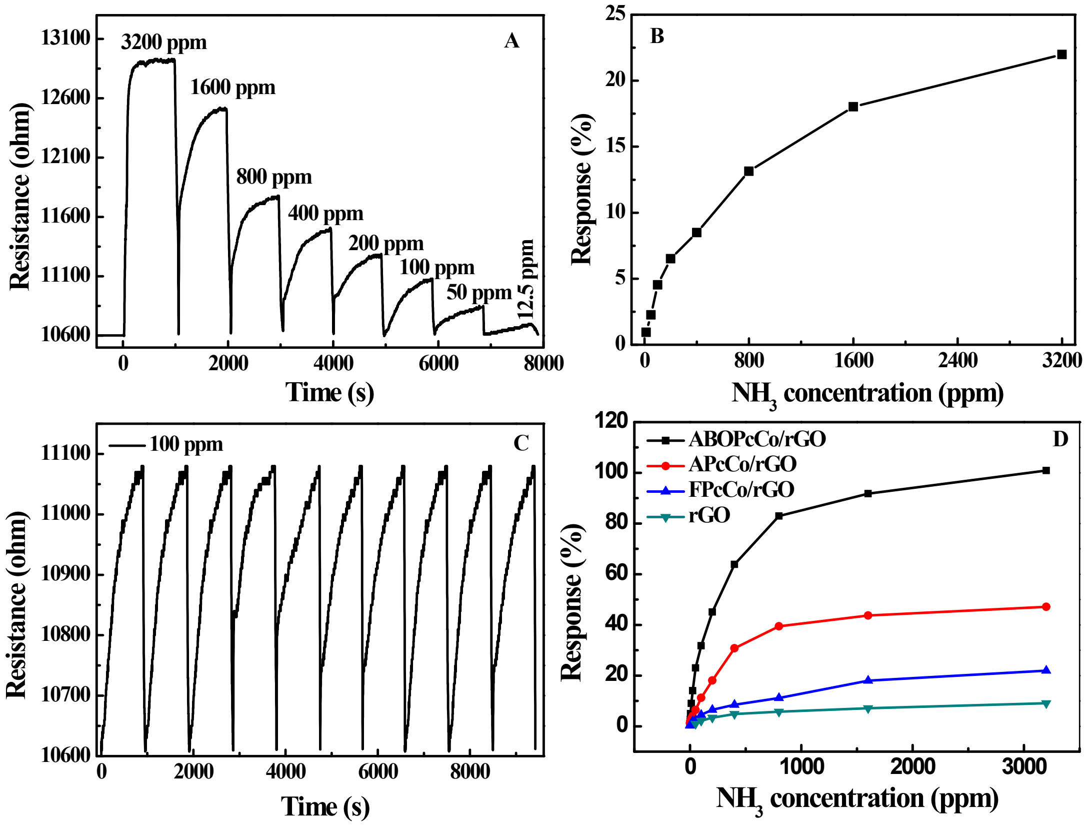

Fig. S8 Resistance of (A) FPcCo/rGO hybrid sensor upon exposure to varying concentrations of NH_3; (B) relationship of the response of FPcCo/rGO hybrid sensor to the concentration NH_3; (C) ten sensing cycles of FPcCo/rGO hybrid sensor to 100 ppm NH_3 at 28°C; (D) relationship of the response of PcCo/rGO hybrid sensors to the concentration NH_3.

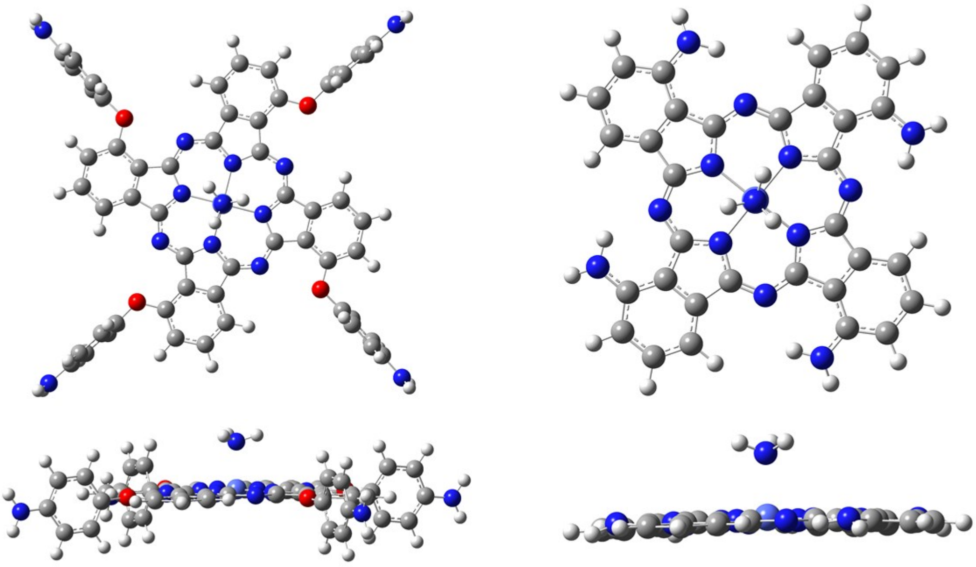

ABOPcCo-NH_3 APcCo-NH_3

Fig. S9. Optimized structures of NH_3-PcCos with (A) top and (B) side views.

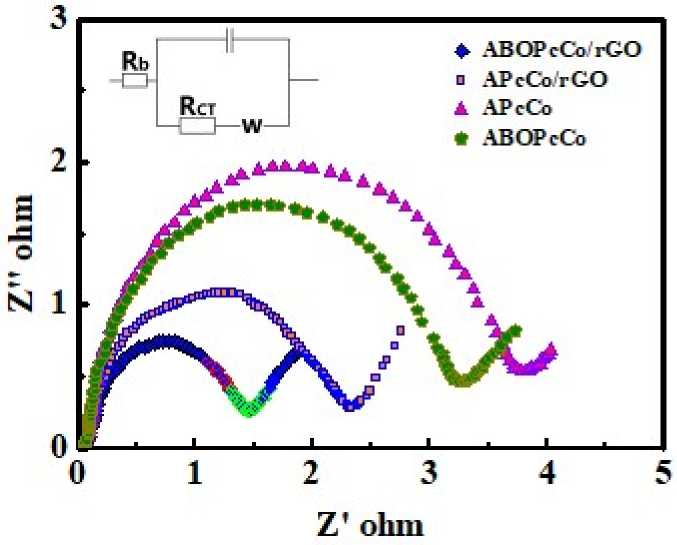

Fig. S10. EIS measurements of ABOPcCo, APcCo, ABOPcCo/rGO and APcCo/rGO sensors.

Table S3. The values of Rct calculated through the impedance spectra using ZSimpWin software.

Sample	$R_{ct}(\Omega)$
ABOPcCo/rGO	1.45
APcCo/rGO	2.34
ABOPcCo	3.35
APcCo	3.82

References

1. C. F. Hua, Y. Y. Shang, Y. Wang, J. Xua, Y. J. Zhang, X. J. Li, A. Y. Cao, A flexible gas sensor based on single-walled carbon nanotube-Fe2O3 composite film, Appl. Surf. Sci. 405 (2017) 405–411.

2. Y. L. Wang, J. Liu, X. B. Cui, Y. Gao, J. Ma, Y. F. Sun, P. Sun,F. M. Liu, X. S. Liang, T. Zhang, G. Y. Lu, NH_3 gas sensing performance enhanced by Pt-loaded on mesoporousWO_3, Sens. Actuators B 238 (2017) 473–481.

3. F. Shao, F. Hernández-Ramíreza, J. D. Prades, C. Fàbrega, T. Andreu, J. R. Morante, Copper (II) oxide nanowires for p-type conductometric NH_3 sensing, Appl. Surf. Sci. 311 (2014) 177–181.

4. M. Das, D. Sarkar, One-pot synthesis of zinc oxide - polyaniline nanocomposite for fabricationof efficient room temperature ammonia gas sensor, Ceramics International 43 (2017) 11123–11131.

5. L. L. Wang, H. Huang, S. H. Xiao, D. P. Cai, Y. Liu, B. Liu, D. D. Wang, C. X. Wang, H. Li, Y. R. Wang, Q. H. Li, T. H. Wang, Enhanced Sensitivity and Stability of Room-Temperature NH_3 Sensors Using Core−Shell CeO_2 Nanoparticles@Cross-linked PANI with p−n Heterojunctions, ACS Appl. Mater. Interfaces. 6 (2014) 14131−14140.

6. X. Li, Y. Gao, J. Gong, L. Zhang, L. Y. Qu, Polyaniline/Ag Composite Nanotubes Prepared through UV Rays Irradiation via Fiber Template Approach and their NH3 Gas Sensitivity, J. Phys. Chem. C 113 (2009) 69-73。

7. J. W. Han, B. Kim, J. Li, M. Meyyappan, A carbon nanotube based ammonia sensor on cellulose paper, RSC Adv. 4 (2014) 549-553.

8. L. K. Randeniya, P. J. Martin, A. Bendavid, J. McDonnell, Ammonia sensing characteristics of carbon-nanotube yarns decorated with nanocrystalline gold, Carbon 49 (2011) 5265-5270.

9. Z. P. Liu, G. M. Liao, S. Y. Li, Y. Y. Pan, X. Y. Wang, Y. Y. Weng, X. H. Zhang and Z. H. Yang, Efficient encapsulation of conducting polyaniline chains inside carbon nanotubes: a new strategy to prepare endohedral CNT materials, J. Mater. Chem. A 1(2013) 13321-13327.

10. R. Ghosh, A. Midya, S. Santra, S. K. Ray, P. K. Guha, Chemically Reduced graphene Oxide for Ammonia Detection at Room Temperature, ACS. Appl. Mater. Interfaces 5 (2013) 7599-7603.

11. F. Yavari; E. Castillo; H. Gullapalli; P. M. Ajayan; N. Koratkar. High sensitivity detection of NO2 and NH3 in air using chemical vapor deposition grown graphene. Appl. Phys. Lett. 100 (2012) 203120-203120-4.

12. R. Kumar, N. Kushwaha, J. Mittal, Superior, rapid and reversible sensing activity of graphene-SnOhybrid film for low concentration of ammonia at room temperature, Sens. Actuators B 244 (2017) 243–251.
